# A Case of Alport Syndrome with Posttransplant Antiglomerular Basement Membrane Disease despite Negative Antiglomerular Basement Membrane Antibodies by EIA Treated with Plasmapheresis and Intravenous Immunoglobulin

**DOI:** 10.1155/2013/164016

**Published:** 2013-12-02

**Authors:** Sumiko I. Armstead, Thomas Hellmark, Jorgen Wieslander, Xin J. Zhou, Ramesh Saxena, Nilum Rajora

**Affiliations:** ^1^Division of Nephrology, Department of Internal Medicine, University of Texas Southwestern Medical Center at Dallas, Dallas, TX 75390, USA; ^2^The Kidney Research Lab, Lund University, Lund, Sweden; ^3^Department of Pathology, Baylor University Medical Center at Dallas, Dallas, TX 75246, USA; ^4^Renal Path Diagnostics, Pathologists BioMedical Laboratories, Lewisville, TX 75067, USA

## Abstract

Posttransplant antiglomerular basement membrane (anti-GBM) disease occurs in approximately 5% of Alport patients and usually ends in irreversible graft failure. Recent research has focused on characterizing the structure of the anti-GBM alloepitope. Here we present a case of a 22-year-old male with end-stage renal disease secondary to Alport syndrome, with a previously failed renal allograft, who received a second deceased-donor kidney transplant. Six days after transplantation, he developed acute kidney injury. The serum anti-GBM IgG was negative by enzyme immunoassay (EIA). On biopsy, he had crescentic glomerulonephritis with linear GBM fixation of IgG. With further analysis by western blotting, we were able to detect antibodies to an unidentified protein from the basement membrane. This patient was treated with plasmapheresis twice per week and monthly intravenous immunoglobulin (IVIG) for a total of five months. At the end of treatment, these unknown antibodies were no longer detected. His renal function improved, and he has not required dialysis. We conclude that anti-GBM disease in patients with Alport Syndrome may be caused by circulating antibodies to other components of the basement membrane that are undetectable by routine anti-GBM EIA and may respond to treatment with plasmapheresis and IVIG.

## 1. Introduction

 Alport syndrome is a genetic disorder caused by mutations in COL4A3, COL4A4, and COL4A5 genes, impairing assembly of type IV collagen. Most cases are inherited in an x-linked pattern, although some cases are autosomal recessive and autosomal dominant [1–3]. Anti-GBM nephritis is usually associated with the presence of circulating IgG antibodies to the noncollagenous domain of alpha 3(*α*_3_) Type IV collagen, manifesting as linear IgG deposition along the GBM on immunofluorescence staining. These patients develop hematuria, proteinuria, and end-stage renal disease. Therapy is unsatisfactory and usually ends in graft failure [4–9]. When patients with Alport syndrome receive renal transplants, posttransplant anti-GBM nephritis occurs in 3–5% of patients [4, 5, 7]. Here we describe a case of Alport syndrome with development of posttransplant anti-GBM nephritis with negative anti-GBM antibodies by enzyme immunoassay (EIA) who was found to have circulating antibodies to another epitope at the noncollagenous region of type IV collagen. The patient was treated with plasmapheresis and IVIG and responded excellently with preservation of renal allograft function.

## 2. Case Report

A 22-year-old male with ESRD secondary to Alport syndrome presented for deceased donor kidney transplantation. The patient had previous kidney transplantation at age of 8 years and had acute T-cell mediated rejection 12 years later, requiring initiation of peritoneal dialysis. His other diagnoses included hypertension and hearing loss. His medications were prednisone, epoetin, potassium chloride, nephrovitamin, sevelamer, lisinopril, amlodipine, carvedilol, and paricalcitol. His mother and maternal grandfather also have Alport syndrome. 

 At presentation, he had no symptoms of infection or cardiovascular disease. The temperature was 37.5°C, blood pressure 120/70 mmHg, pulse 78 beats per minute, respiratory rate 16 breaths per minute, and oxygen saturation 100% on room air. His abdomen was nontender, and his exit site was perfect. The remainder of his physical exam was normal. His calculated panel reactive antibody was 86%. His BUN was 32 mg/dL, creatinine was 20.88 mg/dL, and serum bicarbonate was 30 mmol/L. The other electrolytes and complete blood count were unremarkable. Chest radiograph showed clear lungs and a normal heart and mediastinum.

**Figure 1:**
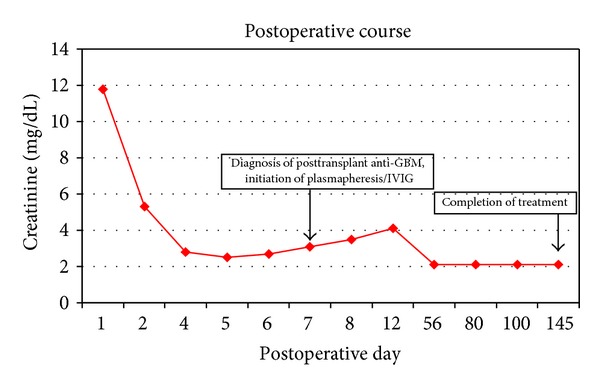
IVIG: intravenous immunoglobulin; GBM: glomerular basement membrane.

The donor was a standard criteria donor with a 4 of 6 human leukocyte antigen mismatch. There was immediate graft function. Postoperative course is depicted in Figure 1. Immunosuppression included thymoglobulin (4 doses), mycophenolate, prednisone, and tacrolimus. His creatinine decreased from 20.88 mg/dL to 2.7 mg/dL by postoperative day (POD)6. On POD7, he developed gross hematuria and acute kidney injury (creatinine 3.1 mg/dL). Urinalysis revealed specific gravity 1.011, large blood, trace leukocyte esterase, 30 mg/dL protein, >720 red blood cells, 7 white blood cells, and 1 squamous epithelial cell. The serum anti-GBM IgG antibody was 0.3 units using multiplex flow immunoassay performed by the Mayo Clinic (≥1 is positive). The tacrolimus level was 4.1 ng/mL.

**Figure 2:**
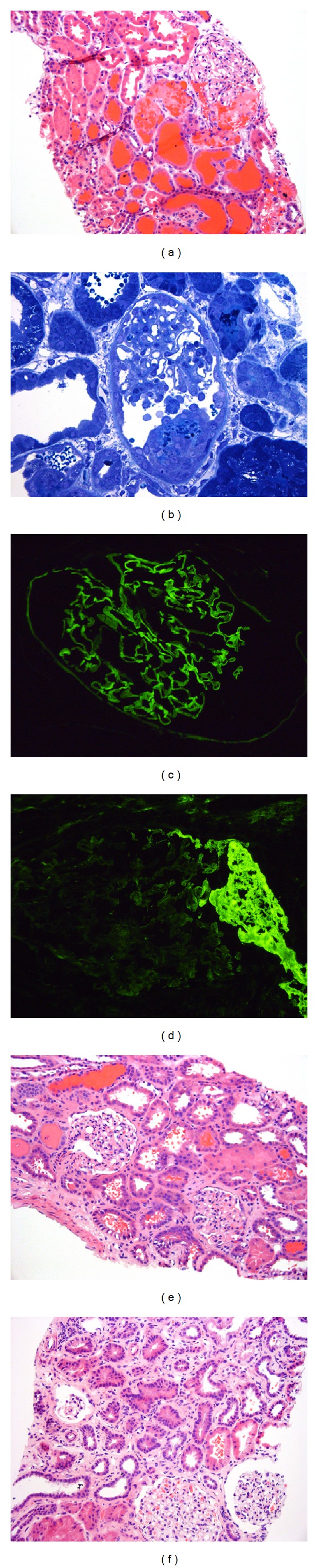
First renal biopsy. (a) Low power view shows a glomerulus with segmental fibrin deposition in the Bowman's space. There is acute tubular injury with many red blood cell casts (H&E ×200). (b) Toluidine blue stained one-micron-thick section reveals a glomerulus with a small cellular crescent. (c) Immunofluorescence staining shows bright linear staining along the capillary walls for IgG. (d) Immunofluorescence staining discloses a segmental fibrinogen staining indicating necrosis/early crescent. Follow-up renal biopsies. (e) Low power view reveals two unremarkable glomeruli with mild tubular injury and few red blood cell casts (2nd biopsy, H&E ×200). (f) Low power view shows focal mild tubular atrophy and interstitial fibrosis. The glomeruli are largely unremarkable (3rd biopsy; H&E ×200).

We performed a renal biopsy. Light microscopy (Figures 2(a) and 2(b)) showed fragments of cortex with 24 glomeruli, none globally sclerotic. One glomerulus revealed a small cellular crescent. Tubules were dilated with flattened epithelial cells, some with red blood cell casts. No tubulitis was present. Immunofluorescence microscopy showed linear staining along the capillary walls for IgG(3+) (Figure 2(c)) and kappa and lambda light chains (1~2+). There was segmental fine granular staining along the capillary walls for C3(2+). One glomerulus disclosed segmental necrosis and a small cellular crescent which stained for fibrinogen (Figure 2(d)). Peritubular capillaries were negative for C4d. Electron microscopy revealed one glomerulus with a cellular crescent. Occasional capillary tufts displayed focal foot process effacement. The diagnosis of posttransplant anti-GBM glomerulonephritis was rendered. The patient was immediately started on plasmapheresis with IVIG. The other immunosuppression was continued. After 5 sessions of plasmapheresis with IVIG, his creatinine was 4.1 mg/dL. 

We then repeated renal biopsy. Light microscopy (Figure 2(e)) showed unremarkable glomeruli without crescents. There was significant acute tubular injury without tubulitis. Immunofluorescence microscopy showed linear staining of glomerular capillary walls with IgG(3+) and kappa and lambda light chains (1~2+). Electron microscopy was largely unremarkable.

Twice weekly plasmapheresis and monthly IVIG were continued for maintenance therapy. He had persistent hematuria; his creatinine decreased to 2.1 mg/dL. Renal biopsy was repeated on POD56. Light microscopy showed unremarkable glomeruli with focal mild tubular atrophy and interstitial fibrosis with patchy lymphoplasmacytic infiltrate (Figure 2(f)). Immunofluorescence microscopy showed linear staining along the capillary walls for IgG with much less intensity (2+) when compared to the two previous biopsies. Peritubular capillaries were negative for C4d. After 3 months of aggressive treatment, IVIG was discontinued, and the frequency of plasmapheresis was decreased to every 2 weeks. Plasmapheresis was discontinued after a total of 5 months; creatinine remained 2.1 mg/dL.

## 3. Immunoassay

**Figure 3:**
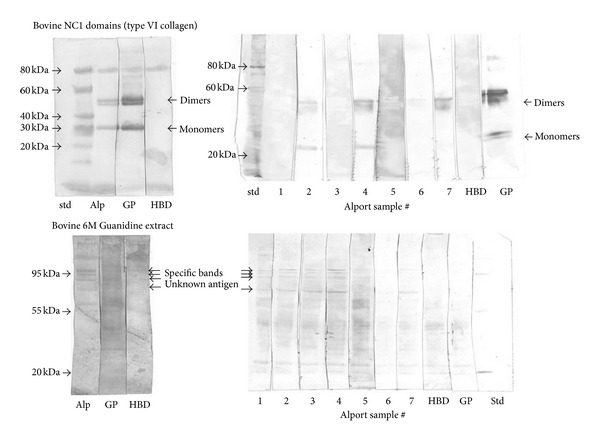
Western blots using purified bovine NC-1 domain of type IV collagen (upper 2 panels) and crude guanidine extract of bovine glomerular basement membrane (lower 2 panels). Sample 1 : 2 months after transplantation. Sample 2 : 1 week after transplantation. Sample 3 : 3 months after transplantation. Sample 4 : immediately prior to the 2nd transplant. Sample 5 : 2 weeks after transplantation. Sample 6 : 7 months after transplantation. Sample 7 : pooled sample of all 6 sera. Std: standard, Alp: pooled Alport sera, GP: goodpasture serum (positive control), HBD: healthy Blood Donor serum (negative control).

As discussed above, the patient's biopsy showed linear deposition of IgG along the GBM, consistent with anti-GBM nephritis; however, we failed to detect circulating anti-GBM antibody by routine EIA. As it is possible that this patient has antibody directed against an epitope on GBM that is not detected by standard EIA, we analyzed reactivity against recombinant noncollagenous (NC) domains of the *α*1, *α*3, and *α*5 chains without finding any specific reactivity. To further explore the reactivity of the autoantibodies, we performed Western blotting using purified noncollagenous 1 domains and 6 M guanidine hydrochloride (GuHCl) extracts from basement preparations as reported in previous studies [5, 6]. We used the patient's serum collected at specific time points: immediately prior to the second transplant and then at 1 week, 2 weeks, 2 months, 3 months, and 7 months after transplantation. Using the type IV collagen NC1 domains as antigen (Goodpasture antigen), despite the negative ELISA results, we found a weak reactivity to some of the samples. It must be stressed that the development time was much longer for the Alport samples (about 1 hour) compared to the Goodpasture sera (2 minutes); the experiment is not quantitative. There were, however, antibodies recognizing an unidentified protein from the 6 M GuHCl extract in each of the samples collected prior to transplantation and at 1 week, 2 weeks, 2 months, and 3 months after transplantation. These antibodies were no longer present at 7 months after transplantation (Figure 3). 

## 4. Discussion

The glomerular basement membrane of patients with Alport syndrome lacks *α*3*α*4*α*5(IV) trimers. Posttransplant anti-GBM nephritis is an allogeneic response to antigens present in the transplanted kidney but absent in the native kidneys of the Alport recipient [7, 10, 11]. In x-linked Alport syndrome, target alloantibodies are to the *α*5(IV) noncollagenous 1 domain primarily [10]. Commercial assays are relatively insensitive to anti-*α*5(IV) antibodies [7]. 

Treatment of posttransplant anti-GBM disease in Alport syndrome remains a challenge. Recent characterization of anti-GBM autoepitopes and alloepitopes revealed differences in the supramolecular structure of the antigen and accessibility of the tissues [11, 12]. The alloepitope is more accessible within NC1 hexamers than the partially sequestered Goodpasture autoepitopes [13, 14]. A better understanding of the alloepitopes in posttransplant nephritis will help to create therapies to restore tolerance to the normal *α*3*α*4*α*5(IV) collagen [15].

In this case, we were unable to detect anti-GBM antibody using EIA, but very faint staining was found in Western blot analysis against bovine NC1 domain of type IV collagen identical to the classical Goodpasture pattern. This may indicate that the titers of circulating antibodies are below the detectable limit of EIA or that there are differences in fine specificity of these antibodies. We did, however, discover antibodies against other unidentified basement membrane protein(s) that were unique to the patient's sera. Based on our results, we cannot say which anti-GBM antibodies are bound to the GBM, but the novel anti-GBM antibodies correlate better with disease progression. Further studies are needed to characterize this antigen.

Current treatment options include methylprednisolone pulse therapy, plasmapheresis, cyclophosphamide, mycophenolate, IVIG, and rituximab, and recent studies indicate bortezomib may be effective as well [7, 16, 17]. Graft failure approaches 90% regardless of the specific therapy used [7]. Here we describe a patient who responded to a combination of plasmapheresis and IVIG. To our knowledge, this combined therapy has not been previously reported in the treatment of posttransplant anti-GBM disease in Alport syndrome. We observed improvement in the patient's renal function, and he has remained dialysis independent for 7 months after transplantation.
